# The Impact of COVID-19 on Migraine: The Patients’ Perspective

**DOI:** 10.3390/life14111420

**Published:** 2024-11-04

**Authors:** Angelo Torrente, Paolo Alonge, Roberta Baschi, Laura Pilati, Vincenzo Di Stefano, Cecilia Camarda, Filippo Brighina, Roberto Monastero

**Affiliations:** 1 Department of Biomedicine, Neuroscience and Advanced Diagnostics (BIND.), University of Palermo, 90127 Palermo, Italy; angelo.torrente@unipa.it (A.T.); paolo.alonge01@unipa.it (P.A.); roberta.baschi@gmail.com (R.B.); laura.pilati.91@gmail.com (L.P.); vincenzo19689@gmail.com (V.D.S.); cecilia.camarda@unipa.it (C.C.); roberto.monastero@unipa.it (R.M.); 2 Neurology and Stroke Unit, P.O. “S. Antonio Abate”, 91016 Trapani, Italy

**Keywords:** COVID-19, pandemic, migraine, CGRP, patient’s report, patient’s point of view, social issue

## Abstract

The COVID-19 pandemic represents a global health phenomenon that will sadly remain part of our history. It had innumerable consequences for society and people’s lives. With different mechanisms, COVID-19 has been pointed out as a factor in the pathophysiology of several secondary disorders or the deterioration of pre-existing conditions. Migraine is a frequent disorder that can be influenced by several conditions, including psychologically stressful conditions or infectious diseases. The purpose of the present study is to gain insight into the influence of COVID-19 on the clinical characteristics of patients with migraine. A self-administrable questionnaire has been developed, asking for migraine features before and after COVID-19 infection. One hundred and two patients who had been infected at least once were included. After COVID-19 infection, 54 reported the worsening of migraine, 45 noticed no variation, and 3 reported an improvement. After the infection, 21 patients changed preventive therapy due to the loss of efficacy of the previous one. The most effective treatments in this subpopulation were gene-related peptide monoclonal antibodies. The presented data confirm that the influence of COVID-19 is heterogeneous in patients with migraine, but new treatments may be effective in controlling the symptoms among those who report a worsening of the disease.

## 1. Introduction

The SARS-CoV-2 pandemic has left a permanent mark on our society and, unfortunately, will forever be remembered by both the scientific and general community. Certainly, social distancing due to the repeated lockdowns caused high levels of distress and had a strong impact on people [1]. On the other hand, the forced isolation brought out the need for new approaches to remote interactions. Thus, many workers started participating in virtual meetings, and so-called smart working spread rapidly. This revolution in work life has had various effects: some workers have felt relief from working from home, while others have been affected by the intrusion of work into their personal life [2]. Regarding lifestyle, people have also begun to use remote means for exercise training, such as telecoaching, through smart technologies [3]. In the medical field, smart working has found less application for physicians, but telemedicine protocols have been developed, particularly for follow-up visits. As for people with medical conditions, the pandemic has affected a huge number of people, especially those with neuromuscular disorders, who have been particularly disadvantaged by reduced physical activity [4].

Migraine is a primary headache characterised by episodes of moderate-to-severe pulsating pain with variable localisation, associated with nausea, light and noise sensitivity, and exercise intolerance [5]. It is a very burdensome disease as people suffer from headache episodes with varying frequency that limit leisure time and work, leading to absenteeism and presenteeism [6,7,8,9]. In addition, even outside the ictal phase, patients with migraine show different cognitive and psychological symptoms compared to non-migraineurs [10,11,12]. Overall, anxiety and depression are among the most frequent psychological conditions that find a strong association with migraine [13,14]. From this point of view, migraine should be considered a persistent disease even if headache attacks occur in episodes. The International Society for Headache disorders classifies migraine as episodic (EM) or chronic (CM) based on its frequency, the last one being diagnosed in case of equal or more than 15 days of headache per month of which at least 8 are with migraine features or lead the patient to acute medications intake. EM is diagnosed when the CM diagnostic criteria are not met [5]. Moreover, some patients experience transitory focal neurological symptoms before or during the pain phase, usually lasting up to 60 min each (i.e., migraine aura). This clinical scenario configures the so-called migraine with aura (MwA) subclassification. On the other hand, patients without aura features can be addressed as migraine without aura (MwoA) [5].

There are several factors influencing migraine frequency and that may lead to chronification (i.e., transition from EM to CM). Regarding lifestyle, it has been found that caffeine misuse, increased body weight, and poor sleep quality can have a detrimental effect on migraine frequency [15,16]. As already mentioned, anxiety and depression are associated with migraine, especially with CM, but the relationship is thought to be bidirectional. Indeed, depression or anxiety may worsen migraine as well [17,18]. Certainly, some factors are internal to the pathophysiology of the disease. There are two main approaches to migraine management: the use of acute medication to stop an ongoing attack and the use of preventive treatment to reduce the recurrence of new attacks. Thus, migraine may also worsen due to ineffective preventive therapy, or due to further medication overuse headache (MOH) [5,19]. Moreover, sudden major life changes (e.g., a divorce, job loss, moving to another town), or traumatic psychological stress may be among the causes of the deterioration in migraine frequency or intensity [20,21]. As for acute migraine treatments, there are general (e.g., non-steroidal anti-inflammatory drugs–NSAIDs), or specific ones, such as triptans or ditans [22]. Even if there are no randomised control trials for opioids, some patient may benefit from their use, especially if suggested from pain management clinics. Patients who experience debilitating migraine attacks for 2 or more days per month might benefit from a preventive treatment [23]. There are several lines of treatments, with classic oral drugs including beta-blockers, antiepileptics, and tricyclic antidepressants. Some patients may benefit from a specific protocol using onabotulinumtoxinA (BoNT-A), which has also been shown to be safe and effective in several aspects of the disease, including quality of life [24,25]. Recently, new injectable monoclonal antibodies acting against the calcitonin gene-related peptide (anti-CGRP mAbs) have been developed with high efficacy and minimal side effects [23,26].

The COVID-19 pandemic also had significant and various effects on headache patients, particularly because of the forced variations in daily habits [27]. Indeed, there has been a sharp reduction in people’s physical activity in conjunction with an increase in time spent in front of digital screens. Furthermore, some patients complained of a worsened quality of sleep during the various lockdowns, thus also affecting migraine headaches. Moreover, the pandemic brought a very high level of psychological distress, which contributed to the deterioration of the clinical conditions of migraine patients [28]. Several chronic pain conditions have been reported after COVID-19 infection and referred to persistent inflammation, immunosuppression, and catabolism syndrome [29]. On the other hand, headache had been reported among the most common neurological symptoms associated with the SARS-CoV-2 infection [30] and could be linked to an increased release of CGRP during this phase [31]. In more detail, it can be found as a prodromal symptom of the disease, as part of the clinical cohort of the infection, or as a long-term sequela after its resolution [32,33,34,35]. The headache reported by COVID-19 patients shows various characteristics that sometimes can resemble a tension-type headache or migraine [36]. In the case of new-onset headache (i.e., in people who never complained of a headache history before COVID-19 infection), several patients who received neurological attention have shown features of rare clinical forms of headache such as new daily persistent headache (NDPH) [5,33,37]. This one is a condition that may develop in the context of a pro-inflammatory cascade that occurs during or after viral infection, including COVID-19 [33,38].

In the past four years, COVID-19 infection has been widely studied, and many patients and clinicians have attributed the onset of various diseases to it. Although a causal relationship may exist in some cases, the COVID-19 pandemic increased people’s health awareness, associating several symptoms with the infection or the vaccination and leading to intensified medical check-ups. In addition, a huge cohort of symptoms has been described as post-acute syndrome (i.e., the so-called long-COVID) [39,40]. The systems involved are various, including the cardiovascular, respiratory, gastrointestinal, musculoskeletal, and nervous system. The infection has been reported to affect negatively previous medical conditions, especially respiratory ones [41]. Moreover, several patients also reported a detrimental effect on previous neurological or psychiatric conditions, such as epilepsy or depression [42]. The last relationship with the long-COVID syndrome should be more studied to demonstrate a pathophysiological causal relationship. Otherwise, it could also be explained by the psychological distress that the post-acute syndrome causes to patients. Several migraine patients have reported a variation, being mostly an increase in headache frequency [43]. Nevertheless, there may be a nocebo effect, as in some cases the deterioration has been reported in the absence of a significant increase in headache days reported by diaries. On the other hand, it is possible that headache modification in quality, location, or response to acute medication. The purpose of the present study is to evaluate the role of COVID-19 infection in the possible evolution in migraine characteristics in a cohort of Italian patients referred to an outpatient headache clinic.

## 2. Materials and Methods

### 2.1. Study Protocol and Questionnaire

The study involved patients referred to the Headache Centre of the University Hospital “Paolo Giaccone” in Palermo, Italy. In mid-January 2024, an e-mail containing a link to a web-based questionnaire was sent to all the available addresses provided by the patients during previous outpatient visits. Three months later, a reminder e-mail was sent to increase the rate of response. The responses between January and June 2024 were later collected and any double reply to the questionnaire was subsequently deleted.

The study was conducted according to the principles of the Declaration of Helsinki and was approved by the Palermo 1 Local Ethics Committee, Palermo, Italy (study code MiCoV2, protocol code 06/2023).

The questionnaire was created using Google Forms, part of the free, web-based Google Docs Editors suite offered by Google (Mountain View, CA, USA). The first item was about the informed consent that the patient had to give before starting the questionnaire. Then, there were general questions regarding demographic characteristics. Subsequent questions focused on the current patient’s diagnosis and status of anti-SARS-CoV-2 vaccines and previous COVID infections. The final questions investigated in detail the influence that the viral disease may have had on the pre-existing migraine condition, according to the patient’s perspective. Among the migraine characteristics studied before and after the infection, monthly migraine days (MMDs), average pain intensity using a numeric rating scale (NRS, going from 1—barely noticeable—to 10—unbearable), and monthly acute medication days (MADs) were asked. Table 1 shows the digital questionnaire administered.

### 2.2. Statistical Analyses

Data obtained from the questionnaire were automatically transferred to a spreadsheet, where some variables were classified as qualitative, and other as quantitative. Continuous variables were reported as mean value ± standard deviation (SD). Comparisons of variables before and after the infection were conducted using Student’s *t*-test. Normality was assessed using the Shapiro-Wilks test. Correlation analyses were conducted using Spearman’s r test. The analyses were performed with JASP, JASP Team (2024). JASP (Version 0.19.0) [Computer software]. A *p* value ≤ 0.05 was considered statistically significant.

## 3. Results

### 3.1. Population

Two hundred and seventy-two patients were reached using the email provided during previous outpatient visits. Of these, only 114 gave informed consent to the study and filled out the questionnaire correctly. Thus, the survey showed a response rate of 41.91%. The responding population of 94 (82.46%) females and 20 (17.54%) males included Caucasian subjects aged 46.02 ± 13.27 years. Among them, 15 (13.16%) were affected by MwA and 99 (86.84%) by MwoA. Six patients (5.2%) had not been vaccinated against SARS-CoV-2; all reported a later infection. Among the vaccinated patients, 12 (11.1%) patients had no documented SARS-CoV-2 infection. Consequently, their remaining data were not considered for further analyses. Therefore, the data from the remaining 102 (89.47%) patients who reported a previous confirmed COVID-19 infection were considered for further analyses. This study population consisted of 84 (82.35%) females and 18 (17.65%) males with an overall age of 44.99 ± 13.11 years. The diagnoses were MwA and MwoA in 13.73% and 86.27%, respectively.

### 3.2. General Results

**Figure 1:**
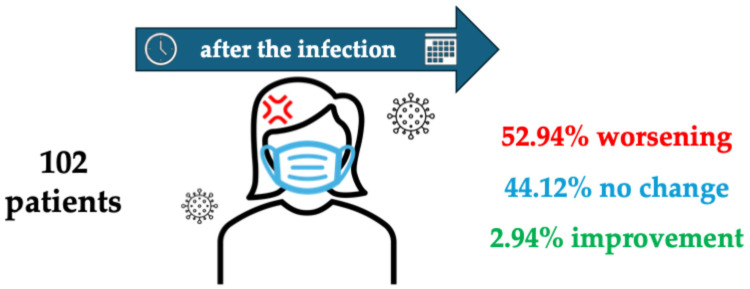
Schematisation of the patients’ report about the influence that COVID-19 caused on migraine perceived characteristics after the infection.

Eighty-seven patients (85.29%) contracted the infection following the vaccination. Most COVID-19 patients (n = 94; 92.16%) experienced only mild symptoms (fever, cough, chills, myalgia), while only 3 (2.94%) developed severe symptoms requiring hospitalisation. Five patients (4.90%) were asymptomatic. During the infection, migraine worsened in half of the patients (n = 51; 50.00%), with one patient reporting an improvement. After the infection, 54 patients (52.94%) reported a deterioration of migraine compared with the pre-infection period, 45 (44.12%) noticed no change, and 3 (2.94%) reported an improvement (see Figure 1). All patients who reported a worsening experienced an increase in migraine frequency. Notably, the effect was most frequently observed immediately after or months after the infection (24 patients each, 23.53%), while subacute worsening (i.e., within one or two weeks) was less common (n = 16; 15.68%). The frequency of migraine increased by 2.6 ± 0.9 MMDs (*p* < 0.001). A similar trend was observed in MADs (+2.5 ± 0.97 days per months, *p* < 0.001). Pain intensity increased slightly but significantly after infection (NRS variation 0.6 ± 0.03 points, *p* <0.001).

### 3.3. Changes in Migraine Characteristics

Twenty-nine patients (28.43%) reported an alteration in the characteristics of their pain. Specifically, after the infection, 20 patients (19.61%) described their pain as pulsating, 11 (10.78%) reported a heavy-headedness/pressing pain, 10 (9.80%) experienced constrictive pain, and 1 (0.98%) described the pain as sharp. Additionally, 6 patients (5.88%) noted a transformation in pain quality, alternating between throbbing and either constrictive or pressing. Nine patients (8.82%) noted a variation in the usual pain location. Finally, 35 patients (34.34%) reported a combination of changes in pain characteristics, headache frequency and/or intensity, and response to medications (either preventive or acute).

### 3.4. Changes in Response to Treatment

Sixty-eight patients (66.67%) received preventive treatment for migraine prior to the SARS-CoV-2 infection; the most common treatment was anti-CGRP mAbs (n = 24; 34.78%), followed by magnesium (n = 14; 20.29%), tricyclic antidepressants (n = 13; 18.84%), antiepileptics (n = 9; 13.04%), beta-blockers (n = 5; 7.25% each), and BoNT-A (n = 3; 2.94% each). After the infection, 21 patients (30.43%) reported a loss of treatment efficacy, and a modification in preventive therapy was suggested to all of them. Among those who switched, 11 patients (52.38%) passed to anti-CGRP mAbs, 3 patients (14.29%) started a tricyclic antidepressant, 2 patients each initiated BoNT-A, magnesium or antiepileptics (9.52% each), and 1 patient (4.76%) switched to beta-blockers. Three patients who were not on preventive medications before the infection started a treatment: one with magnesium, one with anti-CGRP mAbs, and one with BoNT-A. An improvement was reported only by the patient who started anti-CGRP mAb. In contrast, patients who switched prophylaxis experienced a clinical improvement in 57.14% of cases (n = 12). All patients who improved were treated with anti-CGRP mAbs, except for two patients who received tricyclic antidepressants, one who received BoNT-A, and one treated with magnesium. Notably, a single patient previously treated with anti-CGRP mAbs noted an improvement following the infection.

Regarding the acute medications, NSAIDs were the most frequently used (n = 46; 45.10%), followed by triptans (n = 34; 33.33%), acetaminophen (n = 15; 14.70%), and opioids (n = 5; 4.90%). Two patients reported no drug use in the acute phase. After the infection, 31 patients (30.39%) revised the acute therapy, switching mostly to triptans (n = 11; 35.48%) or NSAIDS (n = 10; 32.25%); 7 patients (22.58%) used opioids and 2 (6.45%) acetaminophen. Nineteen of them (61.29%) reported benefit from the variation in therapy, particularly those switching to triptans (10/11, 90.90%); good response rates were obtained even with NSAIDS (6/10, 60%) and opioids (3/7, 42.85%), while none of the patients who switched to acetaminophen reported an improvement. Table 2 summarises all the characteristics of the population who reported a COVID-19 infection.

### 3.5. Long-Term Complications

Most patients did not experience any long-term complication (63.7%); however, 16 patients (15.69%) developed long-term neurological complications, 13 patients (12.74%) showed respiratory complications, 10 (9.80%) autoimmune complications, and 6 patients each experienced endocrine and cardiocirculatory complications (5.88% each).

### 3.6. Correlations

The number of vaccine doses received showed a significant negative correlation with the MMDs (−0.28; *p* = 0.004) and MADs after the infection (−0.22; *p* = 0.020). No significant relationship was found among the other clinical variables (age, sex, type of migraine) and variations after the infection.

## 4. Discussion

Headache is a common symptom in acute respiratory illnesses such as influenza [44]. Similarly, recent studies have shown that COVID-19 had a significant influence on headache patients. First, headache is one of the most common symptoms of the infection [30,34,35]. Moreover, it has been reported that SARS-CoV-2 infection can lead to NDPH, a new-onset headache with heterogeneous characteristics [37,45,46]. The precise pathophysiology of this headache disorder is still not entirely known, but it is thought that COVID-19 may induce a pro-inflammatory cascade that, among other symptoms, may lead to NDPH [47,48,49]. Along the same line, it is not difficult to imagine how, in the same way, COVID-19 may affect a pre-existing headache disorder such as migraine. One study in the literature described how COVID-19 can induce increased level of biomarkers of systemic inflammation. This is thought to be associated with neuroinflammation and subsequent peripheral neuronal sensitisation, underlying the possible worsening of headache even several weeks after the infection [50]. However, patients’ reports are very heterogeneous. In their cohort of patients, Melgarejo et al. described how, among the ones who had been infected with SARS-CoV-2 at least once, about 30% complained of a deterioration of migraine [43]. This population was also significantly more concerned about COVID-19 than that of patients reporting an unmodified migraine. Although some of the subjects reported a detrimental effect of COVID-19 on their perceived clinical conditions, the authors point out that not all the patients showed a real increase in MMDs after the analysis of electronic diaries. Therefore, to explain this contradictory phenomenon, the researchers proposed an infection-related nocebo effect. The data obtained in the present study sample are slightly different from previous studies. About a half of the patients reported a worsening of migraine after the infection, while the others reported an unchanged disease. In general, a significant increase in MMDs and MADs has been found, when looking at all the population data, as well as a slight increase in mean pain intensity. It is noteworthy that there is the exception of three patients who reported an improvement of migraine after COVID-19. This report was rather surprising, perhaps due to the (not investigated) use of corticosteroid drugs during the infection, or a variation in lifestyle habits during and after COVID-19. Indeed, the modification in migraine characteristics could be due to the infection-related lifestyle rearrangement. In fact, some people with mild flu-like symptoms may find a sort of relief in staying at home, away from stressful workplaces, for a few days. On the other hand, several people may experience (and have experienced) isolation due to the infection in a negative way. This psychological stress may affect headache characteristics negatively. The stress should be limited at the time of antigenic nasal swab positivity (and then cease with the end of isolation), but some people may then live with the fear of being infected again. In addition, COVID-19 and its psychological consequences may negatively affect other behavioural domains such as sleep. Sleep and migraine share a dual relationship, influencing each other. Thus, a deterioration in sleep quality may affect migraine negatively, and an increase in migraine frequency with nocturnal headache onset may disturb sleep quality [16]. It has been reported how the pandemic, with social distancing and huge rearrangements in daily routines, has had a serious impact on sleep quality and, consequently, headache [27]. Among the other factors that may have influenced patients’ response to the present questionnaire, there may have been a general disbelief regarding COVID-19 as a disease capable of deteriorating or causing all kinds of complications. It was very common to hear among patients that one symptom or another has started after COVID-19 or, even more frequently, after its vaccine. Although some relationships with COVID-19 may be causative, it should be mentioned that these are patients who have been monitored more closely, so more complications or comorbidities may be discovered compared to the general population. Future studies should involve a larger population of migraine patients and include a multidisciplinary investigation to assess the effects of COVID-19 infection on headache and migraine. In particular, psychological factors and any variation in the daily behavioural routines of migraine subjects during COVID-19 infection will have to be considered.

A large portion (67.65%) of the studied patients were on a preventive treatment before contracting the infection, going from magnesium-based supplements to third- or fourth-line therapies. After the infection, about a third of them (30.43%) reported a reduction in treatment efficacy with a decline in migraine characteristics, so switched to a different preventative. Among those who switched, treatment with anti-CGRP mAbs was the new approach most chosen (52.38%). This means that several patients who perceived a loss in efficacy of previous preventatives were already treatment-resistant patients worthy of anti-CGRP mAbs according to the current regulations [51]. In particular, these ones reported an improvement after starting the new preventative. Serum CGRP levels have been found to be high during COVID-19 infection in inpatients reporting headache, even in the absence of a previous history of migraine, indicating a possible activation of the trigeminal system [31]. This peculiar characteristic may open the hypothesis that with SARS-CoV-2 being a neurotropic virus, it could move from the rhino- and orophararingeal mucosae to the trigeminal afferent neurons and create damage or dysfunction [52,53]. Moreover, the blood CGRP elevation may be more prominent and long-lasting in patients affected by migraine. Consequently, it can disrupt an unstable balance resulting in the need for preventive treatments in patients who did not need them before, or to a variation in the dose or type of medication for the ones already treated. Therefore, therapy with anti-CGRP may be indicated and justified in these patients. Further studies on larger populations are needed to confirm these data. Nevertheless, given the demonstrated safety of these mAbs even during the pandemic [54], they could also be considered in the future as an early line of treatment in migraine patients who complain of a significant headache clinical deterioration after COVID-19 infection.

After the infection, 31 patients (30.39%) of the presented population changed their previous acute therapy, switching mostly to triptans (35.48%), NSAIDs (32.25%), or opioids (22.58%). The highest rates of benefit from the new acute medications was obtained by triptans (90.90% of patients reported a better management of migraine attacks), supporting the use of specific medications compared to general analgesics.

In addition to COVID-19 infection, even anti-SARS-CoV-2 vaccination has been reported to influence migraine frequency [43]. Despite the effects of vaccines not having been investigated in this study, the number of anti-SARS-CoV-2 vaccination shots received by the patients has been recorded. An analysis showed a significant though modest negative correlation of the number of shots with MMDs (−0.28; *p* = 0.004) and MADs (−0.22; *p* = 0.020). Despite the slight effect, this could mean that the vaccination may protect against the post COVID-19 migraine deterioration.

Although this study includes a relatively large group of migraine patients by evaluating different effects of COVID-19 infection on headache characteristics, the study has several limitations. First, caution should be taken in extending the obtained results to the general population. In fact, the study sample was recruited in a headache clinic, with inherent possibility of introducing a selection bias. Second, there may be a misrepresentation of the outpatient population. This is because the questionnaire was sent via email, and some patients may not have been able to access their digital mailbox or fill out the digital questionnaire. In fact, 58.09% of patients did not complete the form, which may mean that elderly patients were not able to complete the form. This aspect may let us reflect on the fact that digital tools are not widely used by all the population despite the recent rearrangements that the pandemic forced upon the society. In addition, the drugs used to treat COVID-19 infection were not investigated. It could have been useful to know whether migraine varied in patients who took one or another drug to treat the infection. Finally, because the onset of the pandemic was 4 years ago, it is possible that patients’ responses were influenced by recall bias. In addition, due to the negative psychological influence of the pandemic and COVID-19, patients may have overestimated their clinical decline in migraine features. Future studies should consider these limitations, including correlations with psychological measures of anxiety, depression, or catastrophising.

## 5. Conclusions

The influence of COVID-19 is relevant for migraine, even if the results differ among patients. Approximately half of them perceive a worsening of their previous condition, while the other half experience no difference or, in rare cases, even experience a slight improvement of disease characteristics. Several aspects of this complicated relationship have yet to be explored and confirmed. Some reports seem to link COVID-19 infection with higher serum levels of CGRP, which could explain a possible better efficacy of anti-CGRP mAbs. This point may in future lead to a rearrangement of the approach to migraine prevention, considering anti-CGRP therapies at earlier lines of therapies. However, it is undeniable that COVID-19 infection psychological and behavioural aspects may have an important role, and these aspects should be taken into account by future studies on this topic. As a last additional factor, even previous vaccination against the virus may have a role in predicting a possible variation in post-infection migraine characteristics.

## Figures and Tables

**Table 1:** Digital questionnaire.

Do you accept that the answers you will give to this form (including your health status) will use anonymously for research studies? * □ Yes □ No
2. Initials * □ ______________
3. Age * □ ______________
4. Sex * □ Male □ Female □ I prefer not to specify
5. Diagnosis * □ Migraine without aura □ Migraine with aura
6. Have you been vaccinated against SARS-CoV-2? * □ Yes □ No
7. If yes, how many shots? □ 1 □ 2 □ 3 □ 4
8. Which vaccine have you got? (select all possible options) □ Pfizer/Biontech □ Moderna □ Astrazeneca □ Johnson&Johnson/Janssen
9. Have you got an infection by SARS-CoV-2 (confirmed by antigenic nasal swab)? * □ Yes □ No
10. If yes, was it before or after the vaccination? □ Before □ After
11. Which SARS-CoV-2 infection symptoms did you develop? □ No symptoms (just positive nasal swab test) □ Mild symptoms (flu-like) □ Severe symptoms (respiratory difficulties, need for hospital admission)
12. During the SARS-CoV-2 infection, how did your migraine change? □ Worsened □ Improved □ Unchanged
13. After the SARS-CoV-2 infection, how did your migraine change? □ Worsened □ Improved □ Unchanged
14. If you noted any change, how long after the infection did you noted the difference? □ Just after the swab test became negative–a few days later □ After 15–20 days □ After a few months
15. How did your migraine change after the infection? (select all possible options) □ The pain became pulsating □ The pain became pressing □ The pain became tightening □ The pain became stabbing □ The pain changed localisation □ The frequency of headache attacks is reduced □ The frequency of headache attacks is increased □ The attacks are stronger □ The attacks are milder □ The attacks last longer □ The attacks are less □ The attacks respond better to acute drugs □ The attacks respond worse to acute drugs □ The preventive therapy has become less effective □ The preventive therapy has become more effective
16. On average, how many migraine days per month did you suffered from before SARS-CoV-2 infection? □ _________
17. On average, how many migraine days did you suffered from after SARS-CoV-2 infection? □ _________
18. On average, how severe were your migraine attacks before SARS-CoV-2 infection? Please indicate a number from 1 (barely noticeable pain) to 10 (unbearable pain) □ _________
19. On average, how severe were your migraine attacks after SARS-CoV-2 infection? Please indicate a number from 1 (barely noticeable pain) to 10 (unbearable pain) □ _________
20. On average, for how many days per month did you use acute medications for migraine attacks before SARS-CoV-2 infection? Please indicate a number from 1 to 30 □ _________
21. On average, for how many days per month did you use acute medications for migraine attacks after SARS-CoV-2 infection? Please indicate a number from 1 to 30 □ _________
22. During the SARS-CoV-2 infection, did you take any medication for migraine prevention? □ Yes □ No
23. If yes, which one? □ Magnesium/other supplement □ Beta-blocker □ Calcium-antagonist □ Antiepileptic drug □ Tricyclic antidepressant □ OnabotulinumtoxinA □ Anti-CGRP monoclonal antibody □ Other: _______________
24. Have you changed preventive therapy because it became ineffective after the SARS-CoV-2 infection? □ Yes □ No
25. If Yes, which new therapy have you started? □ Magnesium/other supplement □ Beta-blocker □ Calcium-antagonist □ Antiepileptic drug □ Tricyclic antidepressant □ OnabotulinumtoxinA □ Anti-CGRP monoclonal antibody □ Other: _______________
26. After the change of preventive therapy, did the migraine improve? □ Yes □ No
27. Which acute medication did you use before the SARS-CoV-2 infection? □ NSAIDs □ Triptans □ Acetaminophen □ Acetaminophen+caffeine □ Opioids
28. Did you change your acute medication after the SARS-CoV-2 infection? □ Yes □ No
29. If Yes, which one? □ NSAIDs □ Triptans □ Acetaminophen □ Acetaminophen+caffeine □ Opioids
30. After the change, has the management of migraine attacks improved? □ Yes □ No
31. Did you experience long-term complications after SARS-CoV-2 infection? □ Neurological complications □ Respiratory complications □ Cardiological complications □ Autoimmune complications □ Endocrinological complication □ Other: __________________

* Mandatory answer.

**Table 2:** Results summary related to the overall population who suffered from COVID-19 infection at least once (n = 102).

Variable	Result
Age	44.90 ± 13.11
Sex	Male: 18 (17.65%)
	Female: 84 (82.35%)
Diagnosis	MwA: 14 (13.72%)
	MwoA: 88 (86.28%)
Vaccinated	96 (94.11%)
Number of doses	1: 6 (5.88%) 2: 26 (25.49%) 3: 63 (61.76%) 4: 7 (6.86%)
Vaccine	Pfizer/Biontech: 58 (56.86%) Moderna: 11 (10.78%) Astrazeneca: 3 (2.94%) Pfizer/Biontech, Astrazeneca: 8 (7.84%) Pfizer/Biontech, Johnson&Johnson: 1 (0.98%) Pfizer/Biontech, Moderna: 23 (22.54%) Moderna, Astrazeneca: 5 (4.90%) Moderna, Johnson&Johnson: 1 (0.98%) None: 6 (5.88%)
Infection before/after vaccination	Before: 15 (14.70%) After: 87 (85.29%)
Infection symptoms	Asymptomatic: 5 (4.90%) Mild symptoms: 94 (92.15%) Severe symptoms: 3 (2.94%)
Migraine changes during infection	No changes: 50 (49.02%) Worsened: 51 (50%) Improved: 1 (0.98%)
Migraine changes after infection	No changes: 45 (44.11%) Worsened: 54 (52.94%) Improved: 3 (2.94%)
Time from infection to symptoms	During the infection—a few days later: 24 (37.50%) After 15–20 days: 16 (25%) After a few months: 24 (37.50%)
Change after the infection	The pain became pulsating 20 (19.61%) The pain became pressing 11 (10.78%) The pain became tightening 10 (9.80%) The pain became stabbing 1 (0.98%) The pain changed localisation 9 (8.82%) The frequency of headache attacks is reduced 3 (2.94%) The frequency of headache attacks is increased 48 (47.05%) The attacks are stronger 34 (33.33%) The attacks are milder 2 (1.96%) The attacks last longer 21 (20.58%) The attacks last less 2 (1.96%) The attacks respond better to acute drugs 3 (2.94%) The attacks respond worse to acute drugs 24 (23.52%) The preventive therapy has become less effective 21 (20.58%) The preventive therapy has become more effective 3 (2.94%)
MMDs before the infection	10.18 ± 8.20
MMDs after the infection	12.85 ± 9.17
Pain intensity before the infection (NRS)	7.19 ± 1.68
Pain intensity after the infection (NRS)	12.85 ± 9.17
MADs before the infection	8.25 ± 6.89
MADs after the infection	10.75 ± 7.85
Preventative before the infection	Magnesium: 14 (13.72%) Tricyclic antidepressants: 13 (13.72%) Antiepileptics: 9 (8.82) Beta-blockers: 5 (4.90%) BoNT-A: 3 (2.94%) Anti-CGRP mAbs: 24 (23.52%) None: 32 (31.37%)
Change in preventive medication after the infection	Magnesium: 3 (2.94%) Tricyclic antidepressants: 3 (2.94%) Antiepileptics: 2 (1.96%) Beta-blockers: 1 (0.98%) BoNT-A: 3 (2.94%) Anti-CGRP mAbs: 12 (11.76%) No change: 78 (76.47%)
Improvement after preventative change	Yes: 13 No: 11
Acute medication before the infection	NSAIDs: 46 (45.09%) Triptans: 34 (33.33%) Acetaminophen: 15 (14.70%) Opioids: 5 (4.90%) None: 2 (1.96%)
Change in acute medication after the infection	NSAIDs: 10 (9.80%) Triptans: 11 (10.78%) Acetaminophen: 2 (1.96%) Opioids: 7 (6.86%) No change: 72 (70.58%)
Improvement after acute medication change	Yes: 19 No: 12

Continuous variables are reported as mean ± standard deviation; qualitative variables are reported as numerosity (percentage). Abbreviations: anti-CGRP mAbs = anti-calcitonin gene-related monoclonal antibodies; BoNT-A = onabotulinumtoxinA; MADs = monthly acute medication days; MMDs = monthly migraine days; MwA = migraine with aura; MwoA = migraine without aura; NSAIDs = non-steroidal anti-inflammatory drugs.

## Data Availability

Data used in this study will be available upon reasonable request made to the corresponding author.
